# The role of microbiomes in cooperative detoxification mechanisms of arsenate reduction and arsenic methylation in surface agricultural soil

**DOI:** 10.7717/peerj.18383

**Published:** 2024-10-30

**Authors:** Nattanan Rueangmongkolrat, Pichahpuk Uthaipaisanwong, Kanthida Kusonmano, Sasipa Pruksangkul, Prinpida Sonthiphand

**Affiliations:** 1 Department of Biology, Faculty of Science, Mahidol University, Bangkok, Thailand; 2 Systems Biology and Bioinformatics Research Laboratory, Pilot Plant Development and Training Institute, King Mongkut’s University of Technology Thonburi, Bangkok, Thailand; 3 Bioinformatics and Systems Biology Program, School of Bioresources and Technology, King Mongkut’s University of Technology Thonburi, Bangkok, Thailand

**Keywords:** Soil microbiome, Arsenic transformation, Arsenic-functional genes, *arsC*, *arsM*

## Abstract

Microbial arsenic (As) transformations play a vital role in both driving the global arsenic biogeochemical cycle and determining the mobility and toxicity of arsenic in soils. Due to the complexity of soils, variations in soil characteristics, and the presence and condition of overlying vegetation, soil microbiomes and their functional pathways vary from site to site. Consequently, key arsenic-transforming mechanisms in soil are not well characterized. This study utilized a combination of high-throughput amplicon sequencing and shotgun metagenomics to identify arsenic-transforming pathways in surface agricultural soils. The temporal and successional variations of the soil microbiome and arsenic-transforming bacteria in agricultural soils were examined during tropical monsoonal dry and wet seasons, with a six-month interval. Soil microbiomes of both dry and wet seasons were relatively consistent, particularly the relative abundance of *Chloroflexi*, *Gemmatimonadota*, and *Bacteroidota*. Common bacterial taxa present at high abundance, and potentially capable of arsenic transformations, were *Bacillus*, *Streptomyces*, and *Microvirga*. The resulting shotgun metagenome indicated that among the four key arsenic-functional genes, the *arsC* gene exhibited the highest relative abundance, followed by the *arsM*, *aioA*, and *arrA* genes, in declining sequence. Gene sequencing data based on 16S rRNA predicted only the *arsC* and *aioA* genes. Overall, this study proposed that a cooperative mechanism involving detoxification through arsenate reduction and arsenic methylation was a key arsenic transformation in surface agricultural soils with low arsenic concentration (7.60 to 10.28 mg/kg). This study significantly advances our knowledge of arsenic-transforming mechanisms interconnected with microbial communities in agricultural soil, enhancing pollution control measures, mitigating risks, and promoting sustainable soil management practices.

## Introduction

Soil is both an important sink and a source of arsenic (As). Mineral soils typically serve as a reservoir for arsenic, while organic soils can function as both a reservoir and a potential source of arsenic (Meharg & Meharg, 2021). The distribution of arsenic in soils varies according to soil particle size fractions and parent materials: however, anthropogenic activities also contribute to elevated arsenic levels in soils (Osuna-Martínez et al., 2021; Zou et al., 2023). Background arsenic concentrations in soils, including agricultural soils, typically range from approximately 2 mg/kg to not more than 20 mg/kg (Xiao et al., 2016; Dunivin, Miller & Shade, 2018; Zhang et al., 2021). However, arsenic concentrations in highly contaminated paddy soils and mining soils may reach levels as high as 800 mg/kg to 18,000 mg/kg (Carrillo-Chavez et al., 2014; Luo et al., 2014).

Arsenic primarily exists in the environment in four different forms, namely arsine (−3), elemental arsenic (0), arsenite (+3), and arsenate (+5), and the common forms found in soils are arsenite and arsenate. Not only inorganic arsenic, but also organic arsenic, such as monomethylarsenic (MMA), dimethylarsenic (DMA), and trimethylarsine (TMAs), can be found in soils (Jia et al., 2013; Ruppert et al., 2013). Due to its carcinogenic properties, arsenic is listed as a first priority hazardous substance by both the US Environmental Protection Agency (EPA) and the Agency for Toxic Substances and Disease Registry (ATSDR) (https://www.atsdr.cdc.gov/SPL/index.html). Acute and chronic exposure to arsenic adversely affects human health. Chronic exposure to arsenic leads to the accumulation of arsenic in specific organs, resulting in various toxic effects such as hepatotoxicity, dermal toxicity, nephrotoxicity, and neurotoxicity (Flora, 2020).

Since the toxicity and mobility of arsenic depend on its oxidation state, microbial arsenic transformations may play crucial roles in controlling the fate and bioavailability of arsenic in the environment. Microorganisms cope with arsenic through energy and detoxification metabolisms (Yan et al., 2019; Irshad et al., 2021). Four major arsenic-transforming pathways are arsenite oxidation, dissimilatory arsenate reduction, detoxification arsenate reduction, and arsenic methylation. Arsenite oxidation, the respiratory oxidization of arsenite to arsenate, is encoded by the *aio* operon. The *aio* operon is composed of the *aioA* and *aioB* genes, which respectively encode the large and small subunits of arsenite oxidase (Lett et al., 2012). Dissimilatory arsenate reduction is catalyzed by the *arr* operon, consisting of the *arrA* and *arrB* genes which respectively encode the large catalytic and small subunits of respiratory arsenate reductase (Saltikov & Newman, 2003). Various bacterial taxa have the ability to utilize arsenate as a final electron acceptor to support their growth. Microorganisms are also able to perform arsenate reduction through a detoxification process which is encoded by the *ars* operon, including the *arsC*, *arsB* and *acr3* genes (Rosen, 2002). Detoxification arsenate reduction involves the *arsC* gene, encoding a small cytoplasmic arsenate reductase, and this process allows various electron donors to complete the reaction (Rosen, 2002; Anderson & Cook, 2004). The *arsC* genes can be divided into two groups: *arsC* (grx) and *arsC* (trx) which use glutaredoxin and thioredoxin as an electron source, respectively. The evolution of the *arsC* gene expanded the spectrum of arsenic resistance, enabling bacteria to detoxify not only arsenite but also arsenate, thereby enhancing their detoxification capabilities (Yan et al., 2019). Arsenic methylation is driven by the *arsM* gene, encoding arsenite S-adenosylmethionine methyltransferase (Qin et al., 2006). This particular enzyme transfers methyl groups from S-adenosylmethionine to arsenite to produce volatile TMAs as the end product. Key arsenic functional genes commonly used as molecular markers to examine the abundance and diversity of arsenic-transforming bacteria in various environments are *aioA*, *arrA*, *arsC*, and *arsM* (Sun et al., 2004; Quéméneur et al., 2010; Jia et al., 2013; Mirza et al., 2017). Gaining a comprehensive understanding of arsenic biotransformation pathways is crucial for assessing the microbial remediation potential and ensuring sustainable soil management practices.

While arsenic-functional genes and their associated microbial taxa in soils, especially in paddy fields, have been previously investigated (Xiao et al., 2016; Zhang et al., 2021; Zhou et al., 2022), the findings have shown both variations and similarities across different soil types. This is possibly because of different environmental factors across locations and a high proportion of uncultured arsenic-transforming microorganisms existing in soils. Biogeography of arsenic-functional genes revealed that according to detection of various genes in different proportions across soils, the composition of microbial communities plays a role in determining local arsenic toxicity and biogeochemistry (Dunivin, Yeh & Shade, 2019). Moreover, microbial arsenic transformations could have an impact on the arsenic biogeochemical cycle in soils with low levels of arsenic. A previous study suggested a high potential for arsenic biotransformations in paddy soils where arsenic concentrations were below 15 mg/kg (Xiao et al., 2016). Arsenic-resistant bacteria were also isolated from soils with 2.58 mg/kg (Dunivin, Miller & Shade, 2018), and a global survey of arsenic genes revealed that the proportions of arsenic genes varied across the soils, indicating the importance of local soil microbiomes in arsenic transformations (Dunivin, Yeh & Shade, 2019). The use of omics technologies and computational analysis has significantly advanced our understanding of arsenic-transforming mechanisms and the microbial response to various levels of arsenic. Consequently, this study employed high-throughput sequencing and PCR-based approaches, targeting both 16S rRNA and arsenic-functional genes, to identify arsenic-transforming pathways in agricultural soil. The objectives of this study were to investigate temporal and successional variations of the soil microbiome and arsenic-transforming bacteria in agricultural soils of the monsoonal tropics during the dry and wet seasons, and to unveil the microbial mechanisms that control arsenic transformations. Identification of arsenic-transforming pathways coupled with analyses of microbial communities will provide in-depth knowledge of the arsenic biogeochemical cycling in sustaining soil quality.

## Materials and Methods

### Sampling site characteristics and sample collection

The sampling site was an agricultural area located in Samphran District, Nakhon Pathom Province, Thailand. The land is mainly used for banana cultivation. Chemical fertilizers and pesticides are applied once per month. The study area is surrounded by factories. According to Thai regulations, the arsenic concentration in soil within residential areas and agricultural areas should not exceed 6 and 25 mg/kg, respectively. Since the study area is located in a residential zone and has the potential for arsenic contamination due to anthropogenic activities, an investigation into arsenic genes associated with the soil microbiome was conducted. Surface soil samples (0–15 cm depth) were collected from three plots across the study area. For each plot, approximately 100 g of surface soil were randomly collected from three locations. In order to make a composite sample, the collected soil samples from three locations from each plot were subsequently pooled and mixed on site. Each composite sample was subsequently analyzed for soil properties. The samples were collected in plastic bags and kept on ice during transportation. The soil samples were collected in February (T1) and August (T2) 2021, respectively representing soils during both the dry and wet seasons.

### Analysis of soil properties

Soil samples were also collected for the analysis of soil properties which were conducted by Central Laboratory (Thailand) Co., Ltd., according to standard protocols. Soil pH and moisture were analyzed according to the method of analysis of chemical fertilizer B.E. 2559 method 1.02.01 and method 1.04.01, respectively. Total organic carbon (TOC) was measured according to the manual on organic fertilizer analysis, APSRDO. Total nitrogen (TN) and total phosphorus (TP) were analyzed by an in-house method based on AOAC (2019). Arsenic (As) and cadmium (Cd) concentrations were respectively analyzed by inductively coupled plasma optical emission spectroscopy (ICP-OES) and inductively coupled plasma mass spectrometry (ICP-MS), according to an in-house method based on AOAC (2019). Copper (Cu) and zinc (Zn) concentrations were analyzed by ICP-OES, according to an in-house method based on official methods of analysis of fertilizers, Japan (1987). The concentrations of arsenic, cadmium, copper, and zinc were analyzed due to their common prevalence in polluted soils and their toxicity to impacted living organisms (Gong et al., 2020). Soil texture was analyzed by mechanical analysis through the pipette method (Olmstead, Alexander & Middleton, 1930).

### DNA extraction

Total genomic DNA was extracted using a DNeasy PowerSoil Pro Kit (Qiagen, Hilden, Germany), following the manufacturer’s protocols. For each sample, multiple soil samples were separately extracted. The quantity and quality of extracted DNA were estimated by a NanoDrop™ One spectrophotometer (Thermo Fisher Scientific, Waltham, MA, USA) and agarose gel electrophoresis. The extracted DNA from each sample were subsequently pooled and used for downstream processes.

### Analysis of 16S rRNA gene sequencing

Library construction and sequencing of the V3–V4 hypervariable regions of the bacteria 16S rRNA gene were prepared using primers 341F and 806R. Triplicate samples were conducted for each sample (T1_1, T1_2, T1_3 and T2_1, T2_2, T2_3). The 16S rRNA gene libraries and sequencing were prepared by Novogene Co., Ltd. (Beijing, China), using an Illumina NovaSeq 6000 platform, following the manufacturer’s protocol. The Illumina platform generated 250 bp paired-end raw reads. Raw data of 16S rRNA gene amplicon sequences were deposited in the Sequence Read Archive (SRA) under the Bioproject number PRJNA980983.

For the data preprocessing step, the characteristics of the raw sequences (length, quantity, and quality score) were evaluated using FastQC version 0.11.8 (Andrews, 2010). Adapter sequences, including barcode and primer sequences, were subsequently trimmed using Cutadapt version 3.5 (Martin, 2011). The minimum length of the trimmed sequences was 200 bp. Amplicon sequence variants (ASVs) were identified using the Divisive Amplicon Denoising Algorithm 2 (DADA2) (Callahan et al., 2016). All analyzed sequences were truncated at 223 bp for forward reads and 219 bp for reverse reads. A classifier was generated for taxonomic assignment with minimum and maximum length of 380 bp and 470 bp, respectively. Taxonomic information was assigned to all ASVs based on the SILVA 138 SSU database (Quast et al., 2013). ASVs assigned as neither *Bacteria* nor *Archaea* were removed. Before the analyzed ASVs were normalized, ASVs with doubletons sequences were eliminated. Alpha rarefaction curves were conducted and alpha diversity indices (*e.g.*, Chao1 and Simpson’s index) were calculated. All the 16S rRNA gene analyses were conducted using the Quantitative Insights Into Microbial Ecology2 (QIIME2) software version 2021.8 (Bolyen et al., 2019). To determine significant difference of microbial taxa between the dry and wet seasons, a two-tailed test was conducted (*p*-value <0.05). Principal Coordinates Analysis (PCoA) based on Bray–Curtis dissimilarities were generated by QIIME2 version 2021.8 and visualized by the RStudio program. To examine the significant difference between microbial groups, permutational multivariate analysis of variance (PERMANOVA) was conducted using QIIME2 version 2021.8.

### Analysis of arsenic specific gene sequencing

Library construction and sequencing of the arsenic functional genes were prepared using specific primers, targeting the *aioA*, *arrA*, *arsC*, and *arsM* genes (Table S1). The libraries and sequencing were prepared by BTSeq™ Contiguous Sequencing Service, using an Enzymatic Preparation Kit (EP Kit) (Celemics, Korea), following the manufacturer’s protocol. An Illumina MiSeq platform was used to generate 150 bp paired-end raw reads. Raw data of arsenic specific gene amplicon sequences were deposited in the Sequence Read Archive (SRA) under the Bioproject number PRJNA980983.

The qualities of raw sequences (length, quantity, and quality score) were assessed using FastQC version 0.11.8 (Andrews, 2010). Pair-ended reads were joined using the Mothur software (Schloss et al., 2009). The joined sequences were subsequently compared against the previously collected arsenic functional gene sequences from the GenBank database using the blastx tool (Camacho et al., 2009). The merged sequences were evaluated based on the criteria of 80% coverages and 80% similarities. For each gene, the analyzed sequences were clustered into 97% operational taxonomic units (OTUs) (Zhang et al., 2015; Yang et al., 2020) using the CD-HIT program version 4.8.1 (Li & Godzik, 2006). Representative OTU sequences of each gene were selected and verified using the blastn tool (Camacho et al., 2009). The verified OTU sequences were included in phylogenetic analysis. Neighbor-joining trees were constructed using MUSCLE alignment (Edgar, 2004; Tamura, Nei & Kumar, 2004) and were generated with the maximum composite likelihood method with 1,000 bootstrap values, using the MEGA X software (Kumar et al., 2018).

### Analysis of shotgun metagenomic sequencing

Library construction and sequencing of the DNA samples (T1_1 to T1_3 and T2_1 to T2_3) were undertaken by Novogene Co., Ltd. (Beijing, China), using an Illumina NovaSeq6000 platform, following the manufacturer’s protocol. The Illumina platform generated 150 bp paired-end raw reads. Raw metagenomic sequencing data were deposited in the Sequence Read Archive (SRA) under the Bioproject number PRJNA980983.

The length and quality scores of raw reads were assessed using FastQC version 0.11.8 (Andrews, 2010). After removing adapter sequences, the analyzed sequences were trimmed using Trimmomatic version 0.36 (Bolger, Lohse & Usadel, 2014). To obtain high-quality sequences, trimming was performed with a quality score cutoff of Phred score >= 20 and a minimum length of 100 bp. Obtained clean reads were assembled into contigs using the metaSPAdes software version 3.15.4 (Nurk et al., 2017). The assembly was performed using multiple k-mer lengths (21, 33, 55, and 77), and the k-mer length of 77 was selected for further analysis. The quality of the assembled contigs was assessed using the QUAST tool version 5.2.0 (Gurevich et al., 2013). The qualified contigs were then annotated using Prokka software version 1.14.6 (Seemann, 2014). The taxonomic profiles were created by mapping metagenomic reads against the standard Kraken2 database. Normalization was employed by scaling with the smallest number of total reads. The qualified contigs were aligned against the customized *aioA*, *arsC*, *arrA*, and *arsM* database. To construct a customized database of arsenic-related genes, complete protein sequences of bacteria for the *aioA*, *arsC*, *arrA*, and *arsM* genes were collected from the National Center for Biotechnology Information (NCBI). Subsequently, redundant bacterial sequences were discarded, retaining only the unique ones. To count gene abundance, the annotated contigs were subsequently mapped against the clean reads obtained from data preprocessing using the Bowtie 2 algorithm version 2.2.1 (Langmead & Salzberg, 2012) and the samtools software version 1.16.1 (Li et al., 2009). The abundance of arsenic functional genes was retrieved using the BEDTools software version 2.30.0 (Quinlan & Hall, 2010). The abundance of arsenic functional genes was then normalized using the Fragments Per Kilobase of transcript per Million fragments mapped (FPKM) method (Trapnell et al., 2010). The abundances of each gene across two seasons were compared using a pair *t*-test.

## Results

### Characteristics of agricultural soils

The agricultural soil samples were collected from both the dry (T1) and wet (T2) seasons which were 6 months apart. Although the proportions of sand, silt, and clay varied, the soil texture of both T1 and T2 samples was classified as clay (Table 1). Soil pH suggested that the soil remained neutral throughout the year. The concentrations of arsenic, cadmium, and copper in both soil samples were comparable, while the concentration of zinc was much higher in T1 than T2 (Table 1). Total organic carbon (TOC) and total phosphorus (TP) in both soils were detected at low concentrations, while total nitrogen (TN) was not detected (Table 1). Overall, the soil properties during both seasons were similar, except for the apparently elevated zinc concentration in the dry season.

**Table 1:** Soil properties of the dry (T1) and wet (T2) seasons.

	T1	T2
pH	7.3	7.0
soil texture (sand, silt, and clay)	Clay (1.72%, 34.00%, and 64.28%)	Clay (1.84%, 37.07%, and 61.09%)
arsenic (As) (mg/kg)	10.28	7.60
cadmium (Cd) (mg/kg)	<0.05	0.03
copper (Cu) (mg/kg)	43.90	60.30
zinc (Zn) (mg/kg)	1,503.60	65.81
total nitrogen (TN) (%)	Not detected	Not detected
total organic carbon (TOC) (%)	1.30	1.40
total phosphorus (TP) (%)	0.30	0.30
moisture content (%)	21.10	19.20

### Soil microbiomes of the dry and wet seasons

The 16S rRNA gene sequencing generated 152,505 to 189,101 raw reads per sample, and they were normalized to obtain a total of 82,109 high-quality reads for downstream analysis (Table S2). Rarefaction curves indicated that sequencing depth was optimal (Fig. S1). The numbers of ASVs in T1 and T2 samples were 2,000 to 2,100 and 1,600 to 1,900, respectively. Species richness and evenness of the dry and wet samples were comparable (Table S3). Principal coordinate analysis (PCoA) based on Bray–Curtis distance and PERMANOVA suggested that although soil microbiomes of each season were separately clustered (Fig. S2), they were not significantly different (*p*-value > 0.05). Overall, the soil microbiome was consistent throughout the year.

**Figure 1:**
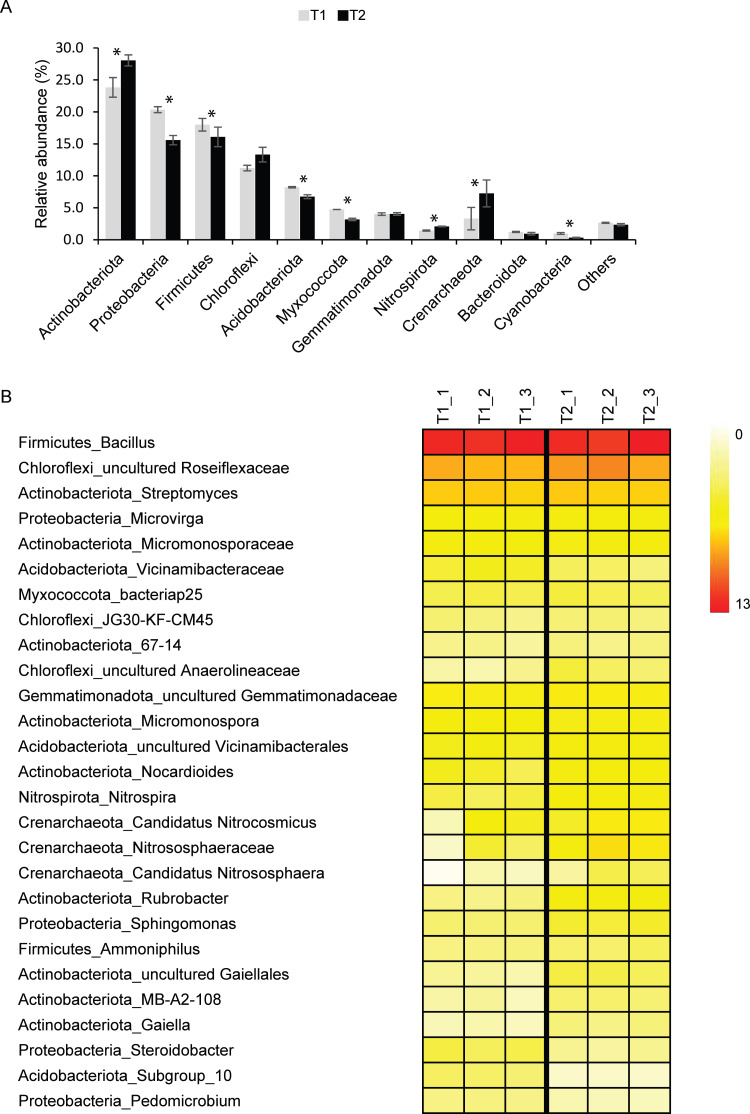
Relative abundance of soil microbiomes of the dry (T1_1, T1_2, and T1_3) and wet (T2_1, T2_2, and T2_3) seasons at the phylum level. Phyla that accounted for less than 1% of the total abundance were merged into the “others” category. Significant differences between the relative abundance of each phylum are indicated by an asterisk (*) (A). Heatmap based on the abundance of microbial taxa at the genus level with more than 1% ASVs at least in one sample. The color intensity indicates the relative abundance of microbial taxa (B).

Although *Archaea* were detected in T1 (∼3%) and T2 (∼7%) samples, *Bacteria* were the dominant domain found in both T1 (∼97%) and T2 (∼93%). The major bacterial phyla found in T1 and T2 samples were *Actinobacteriota*, *Proteobacteria*, *Firmicutes*, *Chloroflexi*, and *Acidobacteriota* (Fig. 1A). Other detected phyla found in T1 and T2 soils were *Myxococcota*, *Gemmatimonadota*, *Nitrospirota*, *Crenarchaeota*, *Bacteroidota*, and *Cyanobacteria*. Although microbial profiles and their relative abundance at the phylum level were similar in both soil microbiomes, statistical analysis indicated that the abundance of *Actinobacteriota*, *Proteobacteria*, *Firmicutes*, *Acidobacteriota*, *Myxococcota*, *Nitrospirota*, *Crenarchaeota*, and *Cyanobacteria* in T1 and T2 were significantly different (*p*-value < 0.05) (Fig. 1A).

For better insight into the dynamics of agricultural soil microbiomes, microbial taxa and their abundance were analyzed at the genus level (Fig. 1B). A total of 27 genera present at greater than 1% of the total microbial abundance in at least one samples were identified. Due to the limitation of the database to identify microbial taxa at the genus level, those in which the genus remained unclassified were identified at the lowest taxonomic rank. Common bacterial taxa highly detected in both T1 and T2 soils were *Bacillus* (∼11%), uncultured_*Roseiflexaceae* (∼6%), and *Streptomyces* (∼4%) (Fig. 1B). *Microvirga* (∼2%) was also consistently present in soils of both the dry and wet seasons. The relative abundance of these taxa in soils from both seasons was not significantly different. Interestingly, the abundance of members of the archaeal phylum *Crenarchaeota*, including *Candidatus* Nitrocosmicus, *Nitrososphaeraceae*, and *Candidatus* Nitrososphaera, was significantly higher in T2 than in T1 (*p*-value < 0.05) (Fig. 1B).

Taxonomic profiles retrieved from metagenomics analysis revealed that top five phyla found in soils from both seasons were *Actinobacteria*, *Proteobacteria*, *Firmicutes*, *Planctomycetes*, and *Bacteroidetes* (Fig. S3). Among the detected genera, *Streptomyces* were the most abundant, followed by *Nocardioides*, *Micromonospora*, *Bacillus*, and *Pseudomonas*, respectively (Fig. S4).

### Communities of arsenic-transforming bacteria

The arsenic-functional genes, *aioA*, *arsC*, *arrA*, and *arsM*, were investigated to study the communities of arsenite-oxidizing bacteria, detoxification arsenate-reducing bacteria, dissimilatory arsenate-reducing bacteria, and arsenic methylation bacteria, respectively. PCR screening of the *aioA*, *arsC*, *arrA*, and *arsM* genes showed that the *arsC* gene was undetectable. Instead of common culture-independent techniques, such as clone library construction, high-throughput sequencing of the *aioA*, *arrA*, and *arsM* genes was employed to explore the communities of arsenic-transforming bacteria. High-throughput sequencing of the *aioA*, *arrA*, and *arsM* genes produced 54,654 to 138,093 raw reads per sample (Table S4).

**Figure 2:**
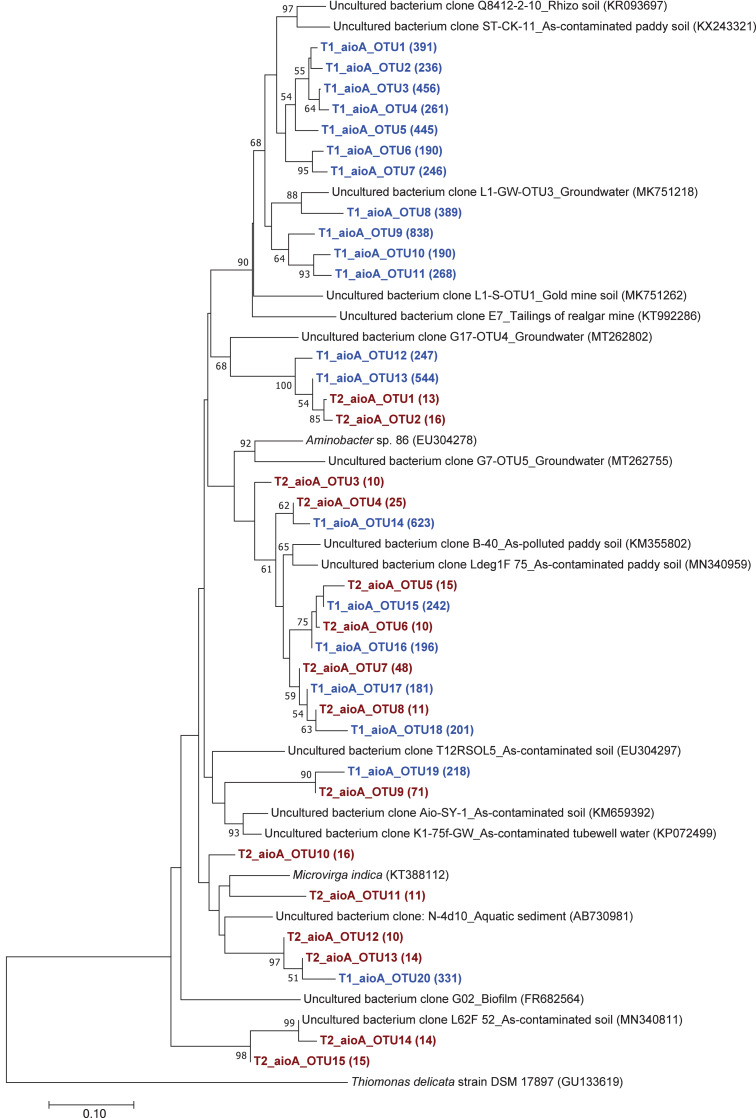
Neighbor-joining phylogenetic tree of the *aioA* sequences. The bootstrap values that are greater than 50% are shown at the branch points. The relative abundances of each OTU are shown in parentheses. The accession numbers of the reference sequences are shown in parentheses.

**Figure 3:**
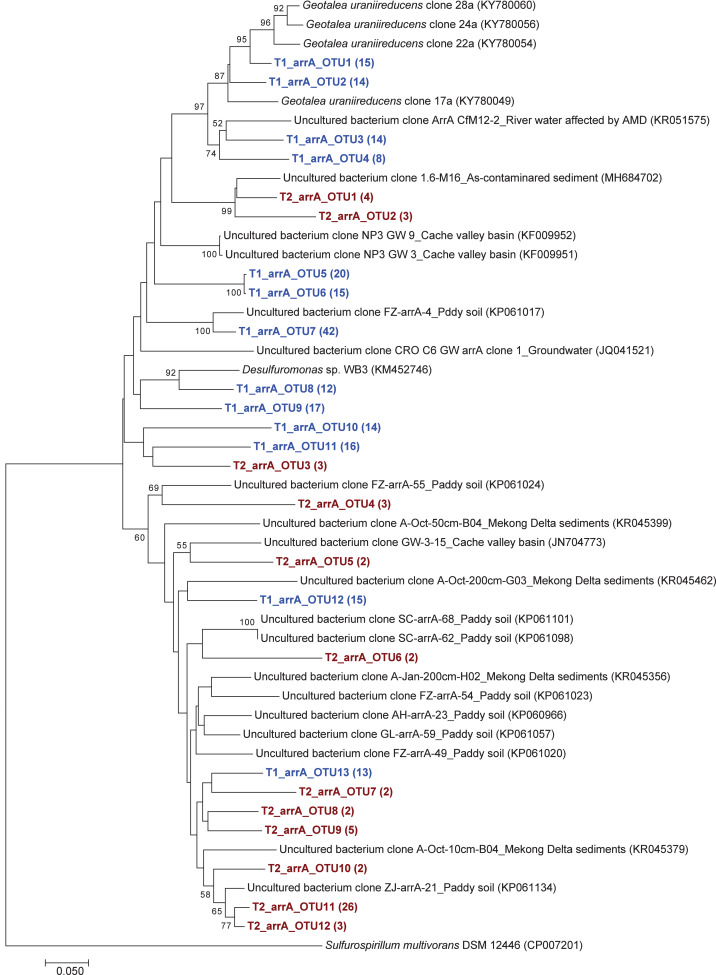
Neighbor-joining phylogenetic tree of the *arrA* sequences. The bootstrap values that are greater than 50% are shown at the branch points. The relative abundances of each OTU are shown in parentheses. The accession numbers of the reference sequences are shown in parentheses.

**Figure 4:**
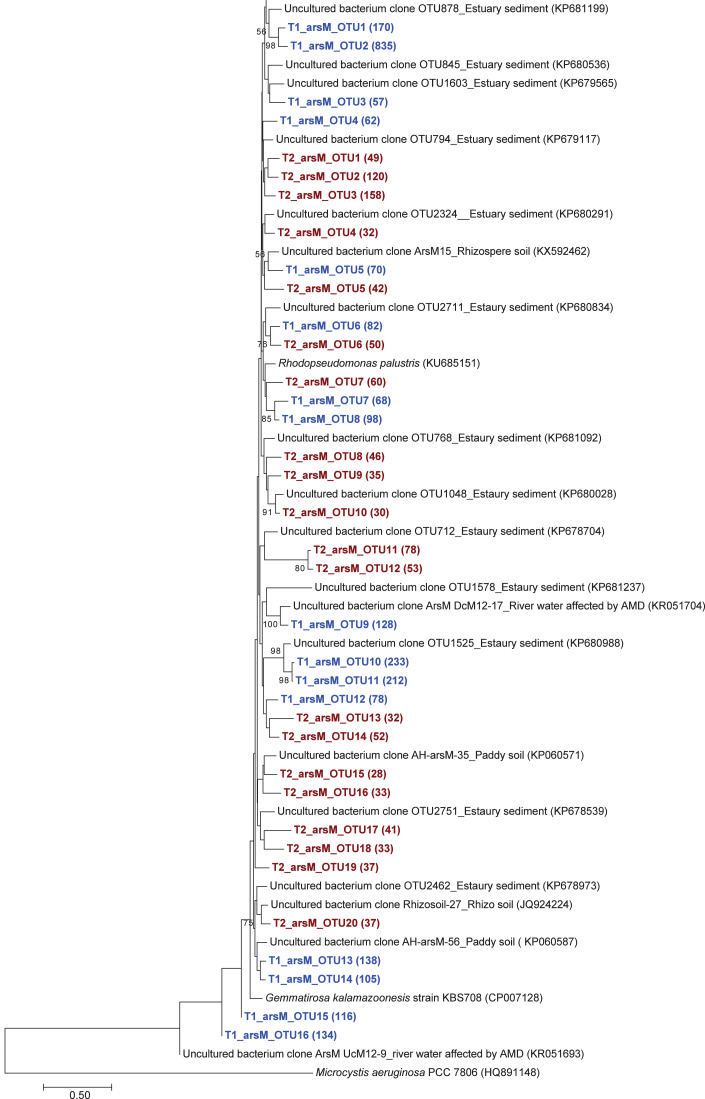
Neighbor-joining phylogenetic tree of the *arsM* sequences. The bootstrap values that are greater than 50% are shown at the branch points. The relative abundances of each OTU are shown in parentheses. The accession numbers of the reference sequences are shown in parentheses.

The phylogenetic tree of the *aioA* sequences revealed that the majority of the *aioA* sequences retrieved from both soil samples were closely related to uncultured *aioA* clones previously recovered from diverse environments, such as arsenic-contaminated soil, groundwater, and aquatic sediment (Fig. 2). While the main retrieved *aioA* sequences from T1 were closely related to uncultured *aioA* clones, a minor portion of those from T2 was closely related to *aioA* sequence belonging to *Microvirga* sp. (Fig. 2). Most analyzed *arrA* sequences from both soil samples were closely associated with uncultured *arrA* clones previously discovered in paddy soils, Mekong Delta sediments, a cache valley basin, and arsenic contaminated sediment (Fig. 3). None of the *arrA* sequences from T2 was associated with known dissimilatory arsenate-reducing bacteria, while those from T1 were closely related to the *arrA* gene belonging to *Geotalea uraniireducens* and *Desulfuromonas* sp. WB3 (Fig. 3). Phylogenetic analysis also demonstrated that the majority of the *arsM* sequences retrieved from both soil samples were closely associated with uncultured *arsM* clones previously reported (Fig. 4). The analyzed *arsM* sequences from T1 were closely related to those of uncultured clones found in estuary sediments, rhizosphere soils, paddy soils, and river water affected by acid mine drainage, while the analyzed *arsM* sequences from T2 were closely associated with those of uncultured clones previously retrieved from estuary sediments, rice rhizosphere soil, and paddy soil. The only known arsenic methylation bacteria closely related to the *arsM* sequences from both soil samples were *Rhodopseudomonas palustris*, which were isolated from soil near a tin mine (Fig. 4).

Overall, the results suggested that the communities of arsenic-transforming bacteria in both soil samples were broadly comparable. The analysis of arsenic-transforming bacterial communities in agricultural soils of both seasons demonstrated that the majority of arsenite-oxidizing bacteria, dissimilatory arsenate-reducing bacteria, and arsenic methylation bacteria found in this study was closely associated with uncultured arsenic-transforming bacteria recovered from various environments.

### Prediction of arsenic-transforming pathways

**Figure 5:**
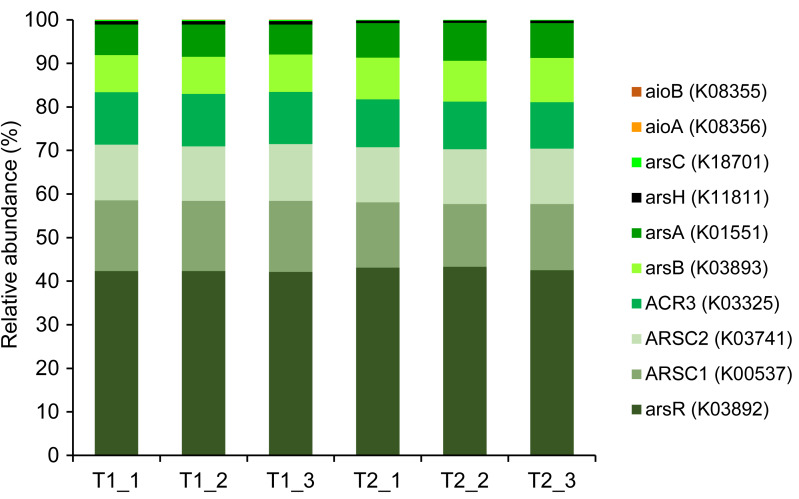
Relative abundance of predicted arsenic-function genes based on 16S rRNA gene sequencing using the PICRUSt2 tool. The KO accession number, as identified in the KEGG database, is provided in parentheses for each arsenic-functional gene.

Based on the resulting 16S rRNA gene sequences, arsenic-functional genes were predicted by the PICRUSt2 software against the KEGG database. Although arsenic functional genes were found in both T1 and T2, they accounted for only ∼0.3% of the total predicted genes. To compare the relative abundance of arsenic functional genes across all samples, only the detected arsenic functional genes were extracted and normalized. The results showed that the proportion of arsenic functional genes in the soils of both dry and wet seasons was relatively consistent (Fig. 5). A paired *t*-test revealed no significant difference (*p*-value >0.05) in the abundances of each arsenic functional gene between T1 and T2, suggesting comparable potential arsenic-transforming pathways in soil across both seasons. A large proportion of the *ars* operon (*e.g.*, *arsC*, *ARSC1*, *ARSC2*, *ACR3*, *arsB*, and *arsA*), responsible for arsenic detoxification mechanisms, was found in the soils analyzed in this study. As for arsenic-functional genes responsible for energy metabolism, the *aioA* and *aioB* genes were detected at very low abundance in both T1 and T2 soils (Fig. 5). Based on the prediction of arsenic pathways through the 16S rRNA gene sequences, arsenic detoxification mechanisms predominated. Since the arsenic cycle in soils is associated with the nitrogen cycle (Feng et al., 2023), genes involved in nitrogen metabolism enzymes were also retrieved (Table S5). Nitrate reduction was highly detected across all soil samples.

**Figure 6:**
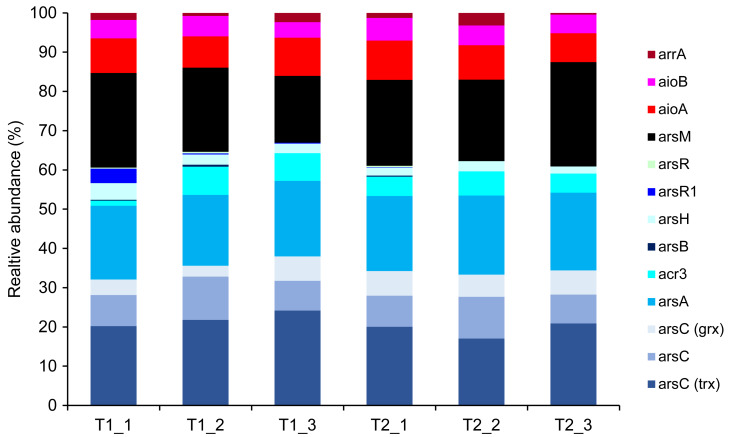
Relative abundance of arsenic-function genes based on shotgun metagenome sequencing using the FPKM method.

Metagenomics sequencing generated a high volume of sequences which were subsequently quality-verified and trimmed of adapter sequences (Table S6). Thirteen arsenic-functional genes in the soil microbiomes were detected and their abundance were normalized by the FPKM method. The results indicated that the overall proportion of arsenic-functional genes in soils of the dry and wet seasons were comparable (Fig. 6). Both arsenic detoxification (*e.g.*, *arsC*, *arsA*, and *acr3*) and metabolism (*e.g.*, *aioA, aioB*, and *arrA*) pathways were identified: however, the relative abundance of arsenic detoxification genes was higher than arsenic metabolism genes. Among the four key functional marker genes, the relative abundance of the *arsC* gene was greatest, followed sequentially by the *arsM*, *aioA*, and *arrA* genes. While the metagenomics analysis of agricultural soil microbiomes identified all four key arsenic-functional genes, the PICRUSt2 prediction tool discovered only the *arsC* and the *aioA* genes (Figs. 5 and 6). Based on shotgun metagenomics analysis, detoxification arsenate reduction and arsenic methylation pathways may cooperatively mitigate arsenic levels in the soil.

## Discussion

### Agricultural soil microbiomes and arsenic-transforming bacteria

The levels of arsenic, cadmium, and copper detected in the soils analyzed in this study were within the range of background levels found in soils elsewhere (Sabet Aghlidi et al., 2020; Liu et al., 2022). Previous studies indicated that zinc concentrations in agricultural soils ranged from 10 to 300 mg/kg (Barber, 1995; Noulas, Tziouvalekas & Karyotis, 2018). However, the extremely high concentration of zinc in the T1 sample cannot be clearly explained due to the lack of sufficient information. The co-occurrence of cadmium, copper, zinc, and arsenic could impact their availability in the soils due to competitive adsorption on soil particles (Lu & Xu, 2009; Gong et al., 2020). However, the specific interactions among these elements are complex, and their availability can vary depending on specific environmental factors and soil conditions. While iron is another element associated with arsenic mobility in soil, a prior study indicated that, in comparison to the clay fraction, both iron and organic matter have a marginal influence on the overall stability and accumulation of arsenic in agricultural soils (Dousova et al., 2016). To assess the impact of these metal elements, including arsenic, on human health, further analysis of their concentrations in banana products is warranted. Low levels of soil nutrients (*i.e.,* TOC, TP, and TN) suggest that they may have been absorbed by crops. Additionally, the absence of nitrogen in both soil samples could be due to microbial nitrification and denitrification (Del Prado et al., 2006), as supported by the high abundance of nitrogen-related functional enzymes, including reductase and ammonia monooxygenase (Table S5). *Nitrospira*, nitrite-oxidizing bacteria, play key roles in nitrification, were also detected in the analyzed soils (Fig. 2A) (Daims & Wagner, 2018).

The concentrations of arsenic in both soil samples analyzed in this study were in the range of those found in background soils (∼2 to 20 mg/kg) (Xiao et al., 2016; Dunivin, Miller & Shade, 2018; Zhang et al., 2021). A previous study showed that coarse sand particles enriched arsenic, while clay particles were the primary source of increased arsenic in soils (Zou et al., 2023). Among different soil particle size fractions, clay harbored the highest abundance of arsenic functional genes, such as the *arrA*, *arsC*, and *arsM* genes (Zou et al., 2023). The high proportion of clay in our analyzed soils may have contributed to the release of arsenic from the soil, favoring the occurrence arsenic-transforming bacteria. Although the arsenic concentrations of the soils in both dry and wet seasons were relatively low, arsenic-transforming bacteria were commonly detected. Although it is complicated to compare taxonomic profiles resulting from the analysis of the 16S rRNA gene and shotgun metagenomics due to differences in technical issues and databases, the top three phyla highly detected by both analyses were *Actinobacteria*, *Proteobacteria*, and *Firmicutes*, respectively (Fig. 1A and Fig. S3). Arsenic-transforming bacteria are ubiquitous in terrestrial environments, especially paddy soils, and arsenate-resistant bacteria affiliated with *Proteobacteria*, *Bacteroidetes*, and *Firmicutes* have been widely isolated from soils without the detection of arsenic (Jackson, Dugas & Harrison, 2005; Xiao et al., 2016; Zhang et al., 2021). Moreover, the major bacterial taxa (*i.e., Actinobacteriota*, *Proteobacteria*, *Firmicutes*) found in this study were highly dominant in banana plantation soils, possibly contributing to the degradation of various organic compounds (Chou et al., 2017; Kaushal et al., 2022).

Agricultural soil microbiomes analyzed in this study were comparable to those previously reported. However, the proportion of dominant phyla varied across soils due to differences in soil characteristics, nutrient loads, and vegetation (Zhao et al., 2019; Liu et al., 2021). The abundance of *Chloroflexi*, *Gemmatimonadota*, and *Bacteroidota* showed no statistical difference between the two seasons, indicating their succession throughout the year. A previous study demonstrated that while the relative abundance of *Gemmatimonadetes* significantly increased, *Chloroflexi* and *Bacteroidetes* in soils were not significantly different during restoration stages (Liu et al., 2021). The phyla *Chloroflexi* and *Bacteroidetes* may constitute a core soil microbiome sustaining soil health.

Common bacterial taxa highly detected in both T1 and T2 soils were *Bacillus*, uncultured *Roseiflexaceae*, and *Streptomyces* (Fig. 1B and Fig. S4). Based on the presence of the *arsC*, *arrA*, *arsM*, and *aioA* genes, members of the genus *Bacillus* have versatile metabolisms in arsenic transformations (Afkar et al., 2003; Huang et al., 2018; Marwa et al., 2019; Mujawar, Vaigankar & Dubey, 2021). *Bacillus* sp. has been suggested for the purpose of arsenic bioremediation (Kabiraj et al., 2022). Although the association between *Roseiflexaceae* and arsenic transformation is not known, this genus is involved in carbon dioxide assimilation and has a positive impact on plant growth (Shi et al., 2020). *Roseiflexaceae* are one of the five keystone taxa found in a long-term fertilized soils used for maize cultivation, and are responsible for enhancing phosphorus flow and preventing the movement of toxic aluminum and manganese from the soil to the crops (Wang et al., 2022). *Streptomyces* sp., previously isolated from the rice rhizosphere, harbors the *arsM* gene, indicating its role in arsenic methylation (Kuramata et al., 2015). In addition, both *Bacillus* sp. and *Streptomyces* sp. were detected at high abundance in healthy banana plantation soils and they are associated with the suppression of banana pathogens (Zhou et al., 2019; Jamil et al., 2022; Kaushal et al., 2022), possibly facilitating banana growth. The genus *Microvirga* was also consistently detected across the soil samples (Fig. 1B and Fig. S4). Members of this genus are distributed across a broad range of environments, including those as diverse as polar soils and hot spring sediments, and they are reported to exhibit both arsenate reduction and arsenite oxidation ability (Tapase et al., 2017; Liu et al., 2020; Zhu et al., 2021). The genus *Nocardioides* was highly abundant, as detected by shotgun metagenomics (Fig. S4). Verification of its *arsC* gene indicated its ability in arsenate reduction (Bagade et al., 2016).

The abundance of *Crenarchaeota*, especially *Candidatus* Nitrocosmicus, *Nitrososphaeraceae*, and *Candidatus* Nitrososphaera, was significantly higher in T2 than T1 (Fig. 1). Members of *Crenarchaeota* are commonly found in soils, playing an important role in ammonia oxidation (Gornish et al., 2020; Behnke et al., 2021). These archaeal taxa potentially drive the nitrogen cycle in the agricultural soils subjected to routine fertilizer application. A previous study showed that among the detected archaeal taxa, *Candidatus* Nitrocosmicus and *Nitrososphaeraceae* were dominant across topsoil (0–10 cm depth) of ten years of restoring eucalypt woodland (Yan et al., 2020). Although *Candidatus* Nitrocosmicus, *Nitrososphaeraceae*, and *Candidatus* Nitrososphaera were also present in the top soils of our dry and wet season samples, they were more abundant in T2 than T1 (Fig. 1B). The increased relative abundance of these archaeal taxa was likely associated with the substantial reduction of zinc concentration in the T2 soil (Table 1). Increasing zinc application to 500 mg/kg in topsoil (0–15 cm depth) inhibited the transcriptional response of archaeal ammonia oxidation (Vasileiadis et al., 2012).

Among the four key arsenic-functional genes representing respective arsenic-transforming bacterial communities, the *arsC* gene was undetectable by PCR amplification possibly due to the limitation of specific primers used in this study. However, the presence of the *arsC* was retrieved from the analysis of 16S rRNA gene sequences through the PICRUSt2 software and shotgun metagenomics (Figs. 5 and 6). It is also possible that the *arsC* gene present in the analyzed soil is likely related to those of uncultured bacteria, making it undetectable using the available specific *arsC* primers.

The phylogenetic trees of *aioA*, *arrA*, and *arsM* revealed that the majority of *aioA*, *arrA*, and *arsM* sequences recovered from the analyzed soils were closely related to uncultured sequences previously found in diverse environments (Figs. 2, 3 and 4). This indicates the ubiquity of arsenic-transforming bacteria in the environment. However, due to the limitation of culture techniques, further exploration is still needed to gain in-depth knowledge of their association with arsenic metabolisms. Our results also showed that a few minor retrieved *aioA* sequences from the T2 sample were closely related to the known *aioA* sequence of *Microvirga* sp. The genus *Microvirga*, previously isolated from polluted soils, is capable of performing arsenite oxidation through the *aioA* gene (Tapase et al., 2017; Tapase & Kodam, 2018). Some minor detected *arrA* sequences from the T1 sample were associated with the known dissimilatory arsenate-reducing bacteria, *Geotalea uraniireducens* and *Desulfuromonas* sp. WB3, which were respectively isolated from paddy field soil and anoxic sediment (Osborne et al., 2015; Qiao et al., 2018). *Rhodopseudomonas palustris* was identified as the sole known arsenic methylation bacterium, exhibiting a close relationship with the *arsM* sequences from both T1 and T2 samples. *Rhodopseudomonas palustris*, well-known arsenic methylation bacterium harboring the *arsM* gene, was commonly found in paddy soils (Xiao et al., 2016; Afroz et al., 2019).

### Potential arsenic-transforming pathways in agricultural soils

The presence of potential arsenic-transforming pathways in agricultural soils were predicted by both the analysis of 16S rRNA gene sequences and by shotgun metagenomics. Both analyses indicated that arsenic detoxification, as evidenced by the significant detection of a large proportion of the *ars* operon, played a pivotal role in driving the arsenic cycle in the agricultural soil (Figs. 5 and 6). In other arsenic-contaminated and uncontaminated soils (arsenic levels < 20 mg/kg) analyzed by metagenomics, arsenic detoxification overcame arsenic energy metabolisms (Xiao et al., 2016; Dunivin, Yeh & Shade, 2019). Based on the identified detoxification arsenic-functional genes, arsenate reduction conferred by the *arsC* gene was a dominant pathway. The *arsC* mechanism is the most extensive-studied arsenic detoxification and resistance. Arsenate exhibited maximal adsorption on clays under low pH conditions, but its adsorption capacity decreased as the pH increased (Goldberg, 2002). Given that arsenate serves as a substrate for arsenate reduction, it could be released from the soils analyzed in this study, enhancing the abundance and activity of the *arsC* gene. The properties of our analyzed soils, including pH and texture, could enhance the availability of arsenate for arsenate-reducing bacteria. The abundance and expression of the *arsC* gene in surface soils were generally high (Luo et al., 2014; Xiao et al., 2016). By analyzing the genome of aerobic arsenate-reducing bacteria isolated from arsenic-contaminated soil, together with the *arsC* gene, the *arsH*, *arsB*, and *arsR* genes controlling the regulation of arsenate reduction were identified (Tian & Jing, 2014). The detoxification arsenate reduction involves the cytoplasmic reduction of arsenate to arsenite by the *arsC* gene. Then, the produced arsenite is transported out of the bacterial cell through a specialized arsenite-pump which were mediated by the *arsA*/*arsB* or *acr3* genes (Singh et al., 2021). The large proportion of *arsC*, *arsA*, *arsB*, and *acr3* genes identified by both the PICRUSt2 program and the FPKM method supported that detoxification arsenate reduction within the soil was an important arsenic-transforming pathway. Cultivation-independent studies proposed that the *arsC* (*grx*) gene was less tolerant to high arsenic concentrations compared to the *arsC* (*trx*) gene, and increased levels of arsenic in a specific area led to a higher prevalence of the *arsC* (*trx*) gene (Escudero et al., 2013; Kurth et al., 2017). However, in this current study, the relative abundance of the *arsC* (*trx*) gene was higher in soils with low arsenic concentrations than the *arsC* (*grx*) gene (Fig. 6). The *arsC* (*trx*) gene is commonly found in Gram-positive bacteria (Mukhopadhyay et al., 2002). The high abundance of the genera *Bacillus* and *Streptomyces*, Gram-positive bacteria, possibly contributed to the higher relative abundance of the *arsC* (*trx*) than the *arsC* (*grx*) genes. The high abundance of *arsR* gene detected by the PICRUSt2 program possibly because the *arsR* genes across various bacterial strains are highly conserved (Xu et al., 1998). Overall results suggest that the detoxification arsenate reduction pathway is widespread in soils, both those with arsenic contamination (88.93–646.61 mg/kg) and those without (less than 15 mg/kg) (Xiao et al., 2016; Zhou et al., 2022).

Metagenomic analysis demonstrated that the second most prevalent arsenic-transforming pathway was arsenic methylation mediated by the *arsM* gene (Fig. 6). Biogeography of soil microbiomes revealed that the *arsM* gene was unexpectedly abundant, especially in cultivation-independent samples (Dunivin, Yeh & Shade, 2019). In paddy soils with low arsenic concentrations (less than 15 mg/kg) and polluted soils with extremely high arsenic concentration (88.93–646.61 mg/kg), arsenic methylation was also identified as the second most prominent process, following detoxification through arsenate reduction (Xiao et al., 2016; Zhou et al., 2022). Previous studies demonstrated that arsenic methylation was active under aerobic, anaerobic, and facultative conditions (Wang et al., 2014). *In silico* analysis also suggested that the *arsM* gene sequences are widespread in the environment and are conserved across various living organisms (Kabiraj et al., 2023). The *arsM* gene catalyzed the conversion of arsenite to volatile methyl arsenic to complete arsenic methylation process (Qin et al., 2006). Consequently, in surface soil, detoxification through arsenate reduction leads to the production of arsenite, which is subsequently methylated by arsenic methylation process. This suggests a cooperative mechanism between detoxification arsenate reduction and arsenic methylation in the surface soil from the agricultural area with low arsenic level. Significant coexistence of the *arsC* and *arsM* genes was previously found in paddy soils (Zhang et al., 2015; Yang et al., 2020). The mechanism of detoxification arsenate reduction and arsenic methylation was also identified in a plant body, primarily in its root (Ma et al., 2016).

The genes involved in methylation and reduction processes exhibited higher abundance compared to those associated with the oxidation process (Figs. 5 and 6). Arsenate is typically more abundant in soils compared to arsenite. Since arsenic methylation genes are commonly found in soils (Dunivin, Yeh & Shade, 2019), they may compete with arsenite oxidation genes for the arsenite produced during arsenate reduction. Additionally, arsenite is more mobile compared to arsenate. The arsenite produced through arsenate reduction could potentially leach into the aquatic environment. Consequently, due to the available arsenic substrate and the prevalence of arsenic methylation genes in soils, both arsenate reduction and arsenic methylation are likely to play roles in arsenic transformations in soils.

Since the abundance of genes mediating nitrate reduction mechanisms was highly detected (Table S5), it suggests the potential for arsenite oxidation coupled with nitrate reduction process. Due to the soil texture, the presence of clay could create anoxic conditions, facilitating the conditions for arsenite oxidation coupled with nitrate reduction. Previous studies suggested that co-occurrence of arsenite oxidation and nitrate reduction was an important process controlling arsenic availability in paddy soils (Li et al., 2019; Feng et al., 2023).

While shotgun metagenomics identified all four arsenic-transforming mechanisms: detoxification arsenate reduction (*arsC*), arsenite oxidation (*aioA*), arsenic methylation (*arsM*), and dissimilatory arsenate reduction (*arrA*), the analysis of 16S rRNA gene sequences found only the first two pathways. The use of shotgun metagenomics allows for broader detection of environmental patterns and variations in the abundance of arsenic-functional genes, as it targets all genes irrespective of primer coverage and specificity. Although the accuracy of PICRUSt2 in predicting microbial functions has improved, it is more suitable for human microbiome than soil microbiome samples (Langille et al., 2013; Sun, Jones & Fodor, 2020). Consequently, some functional genes, including those associated with arsenic, can be overlooked by using the PICRUSt2 prediction tool.

## Conclusions

Microbial arsenic-transforming genes are important in understanding environmental arsenic pathways and evaluating bioremediation potential. Consequently, this study combined high-throughput amplicon sequencing and shotgun metagenomic sequencing to identify potential arsenic-transforming pathways in surface agricultural soils of the dry and wet seasons with a six-month interval. The soil microbiomes showed a relatively consistent composition during both the dry and wet seasons, exemplified by the year-round relative abundance of *Chloroflexi*, *Gemmatimonadota*, and *Bacteroidota*. Among the prevalent bacterial taxa, *Bacillus*, *Streptomyces*, and *Microvirga* were frequently found and potentially exhibited the ability to undergo arsenic transformations. Phylogenetic analyses of the *aioA*, *arrA*, and *arsM* genes suggested that they were closely related to those of uncultured bacteria previously recovered from various environments. Due to the presence of a significant number of uncultured microorganisms in the environment that possess arsenic functional genes, the current selection of primers for detecting these genes is inadequate. Shotgun metagenomic sequencing technology was then utilized to obtain comprehensive information about functional genes across the entire environment. The shotgun metagenome showed that, among the four key functional marker genes, the *arsC* gene was the highest abundance, followed by the *arsM*, *aioA*, and *arrA* genes, respectively. However, due to the limitation of the database, the PICRUSt2 prediction tool identified only the *arsC* and *aioA* genes. Although arsenite oxidation (*aioA*), dissimilatory arsenate reduction (*arrA*), arsenate detoxification reduction (*arsC*), and arsenic methylation (*arsM*) can occur, the latter two detoxification mechanisms are proposed as the key processes in the analyzed soils. Overall, this study indicates that the biogeochemical cycle of arsenic in the surface soil of agricultural areas with low arsenic levels is primarily driven by a cooperative process involving detoxification through arsenate reduction and arsenic methylation. Unlike previous studies that focused primarily on arsenic transformations in high-arsenic environments, this study demonstrates that in surface agricultural soils with low arsenic levels, the biogeochemical cycle of arsenic is predominantly driven by a cooperative process involving detoxification through arsenate reduction and arsenic methylation. Further investigation is needed to understand the activity of arsenic transformations, which could be accomplished through a microcosm study coupled with metatranscriptomic analysis. This study expands our understanding of arsenic-transforming mechanisms, and provides valuable insights that could contribute to sustainable soil management.

##  Supplemental Information

10.7717/peerj.18383/supp-1 Supplemental Information 1 Rarefaction curves of soil samples collected from the dry (T1) and wet (T2) seasons with three replications for each season (T1_1, T1_2, T1_3 and T2_1, T2_2, T2_3)

10.7717/peerj.18383/supp-2 Supplemental Information 2 Principal coordinate analysis (PCoA) based on Bray–Curtis distance matrix of soil samples collected from the dry (T1) and wet (T2) seasons with three replications for each season

10.7717/peerj.18383/supp-3 Supplemental Information 3 Relative abundance obtained from metagenomic analysis at the phylum level of both the dry season (T1_1, T1_2, and T1_3) and wet season (T2_1, T2_2, and T2_3)

10.7717/peerj.18383/supp-4 Supplemental Information 4 Relative abundance obtained from metagenomic analysis at the genus level of both the dry season (T1_1, T1_2, and T1_3) and wet season (T2_1, T2_2, and T2_3)

10.7717/peerj.18383/supp-5 Supplemental Information 5 Arsenic specific primers used in this study

10.7717/peerj.18383/supp-6 Supplemental Information 6 Summary of the 16S rRNA gene sequence reads during quality control assessment

10.7717/peerj.18383/supp-7 Supplemental Information 7 Diversity indexes of soil samples

10.7717/peerj.18383/supp-8 Supplemental Information 8 Summary of the aioA, arrA, and arsM sequence reads during quality control assessment

10.7717/peerj.18383/supp-9 Supplemental Information 9 Abundance of sequences associated with nitrogen metabolism, as determined by 16S rRNA gene sequencing using the PICRUSt2 tool

10.7717/peerj.18383/supp-10 Supplemental Information 10 Summary of the metagenomic sequence reads during quality control assessment

## Additional Information and Declarations

### Data Availability

The following information was supplied regarding data availability: The raw data are available at the Sequence Read Archive (SRA): PRJNA980983.
https://www.ncbi.nlm.nih.gov/bioproject/PRJNA980983/.

## References

[ref-1] Afkar E, Lisak J, Saltikov C, Basu P, Oremland RS, Stolz JF (2003). The respiratory arsenate reductase from *Bacillus selenitireducens* strain MLS10. FEMS Microbiology Letters.

[ref-2] Afroz H, Su S, Carey M, Meharg AA, Meharg C (2019). Inhibition of microbial methylation via *arsM* in the rhizosphere: arsenic speciation in the soil to plant continuum. Environmental Science & Technology.

[ref-3] Anderson CR, Cook GM (2004). Isolation and characterization of arsenate-reducing bacteria from arsenic-contaminated sites in New Zealand. Current Microbiology.

[ref-4] Andrews S (2010). FastQC: a quality control tool for high throughput sequence data. http://www.bioinformatics.babraham.ac.uk/projects/fastqc.

[ref-5] Bagade AV, Bachate SP, Dholakia BB, Giri AP, Kodam KM (2016). Characterization of *Roseomonas* and *Nocardioides* spp. for arsenic transformation. Journal of Hazardous Materials.

[ref-6] Barber SA (1995). Soil nutrient bioavailability: a mechanistic approach.

[ref-7] Behnke GD, Kim N, Zabaloy MC, Riggins CW, Rodriguez-Zas S, Villamil MB (2021). Soil microbial indicators within rotations and tillage systems. Microorganisms.

[ref-8] Bolger AM, Lohse M, Usadel B (2014). Trimmomatic: a flexible trimmer for Illumina sequence data. Bioinformatics.

[ref-9] Bolyen E, Rideout JR, Dillon MR, Bokulich NA, Abnet CC, Al-Ghalith GA, Alexander H, Alm EJ, Arumugam M, Asnicar F, Bai Y (2019). Reproducible, interactive, scalable and extensible microbiome data science using QIIME 2. Nature Biotechnology.

[ref-10] Callahan BJ, McMurdie PJ, Rosen MJ, Han AW, Johnson AJA, Holmes SP (2016). DADA2: high-resolution sample inference from Illumina amplicon data. Nature Methods.

[ref-11] Camacho C, Coulouris G, Avagyan V, Ma N, Papadopoulos J, Bealer K, Madden TL (2009). BLAST+: architecture and applications. BMC Bioinformatics.

[ref-12] Carrillo-Chavez A, Salas-Megchun E, Levresse G, Munoz-Torres C, Perez-Arvizu O, Gerke T (2014). Geochemistry and mineralogy of mine-waste material from a skarn-type deposit in central Mexico: modeling geochemical controls of metals in the surface environment. Journal of Geochemical Exploration.

[ref-13] Chou YM, Shen FT, Chiang SC, Chang CM (2017). Functional diversity and dominant populations of bacteria in banana plantation soils as influenced by long-term organic and conventional farming. Applied Soil Ecology.

[ref-14] Daims H, Wagner M (2018). Nitrospira. Trends in Microbiology.

[ref-15] Del Prado A, Merino P, Estavillo JM, Pinto M, González-Murua C (2006). N_2_O and NO emissions from different N sources and under a range of soil water contents. Nutrient Cycling in Agroecosystems.

[ref-16] Dousova B, Buzek F, Lhotka M, Krejcova S, Boubinova R (2016). Leaching effect on arsenic mobility in agricultural soils. Journal of Hazardous Materials.

[ref-17] Dunivin TK, Miller J, Shade A (2018). Taxonomically-linked growth phenotypes during arsenic stress among arsenic resistant bacteria isolated from soils overlying the Centralia coal seam fire. PLOS ONE.

[ref-18] Dunivin TK, Yeh SY, Shade A (2019). A global survey of arsenic-related genes in soil microbiomes. BMC Biology.

[ref-19] Edgar RC (2004). MUSCLE: multiple sequence alignment with high accuracy and high throughput. Nucleic Acids Research.

[ref-20] Escudero LV, Casamayor EO, Chong G, Pedrós-Alió C, Demergasso C (2013). Distribution of microbial arsenic reduction, oxidation and extrusion genes along a wide range of environmental arsenic concentrations. PLOS ONE.

[ref-21] Feng M, Du Y, Li X, Li F, Qiao J, Chen G, Huang Y (2023). Insight into universality and characteristics of nitrate reduction coupled with arsenic oxidation in different paddy soils. Science of the Total Environment.

[ref-22] Flora SJS, Srivastava S (2020). Preventive and therapeutic strategies for acute and chronic human arsenic exposure. Arsenic in drinking water and food.

[ref-23] Goldberg S (2002). Competitive adsorption of arsenate and arsenite on oxides and clay minerals. Soil Science Society of America Journal.

[ref-24] Gong B, He E, Qiu H, Van Gestel CA, Romero-Freire A, Zhao L, Xu X, Cao X (2020). Interactions of arsenic, copper, and zinc in soil-plant system: Partition, uptake and phytotoxicity. Science of the Total Environment.

[ref-25] Gornish ES, Franklin K, Rowe J, Barberán A (2020). Buffelgrass invasion and glyphosate effects on desert soil microbiome communities. Biological Invasions.

[ref-26] Gurevich A, Saveliev V, Vyahhi N, Tesler G (2013). QUAST: quality assessment tool for genome assemblies. Bioinformatics.

[ref-27] Huang K, Xu Y, Packianathan C, Gao F, Chen C, Zhang J, Shen Q, Rosen BP, Zhao FJ (2018). Arsenic methylation by a novel ArsM As (III) S-adenosylmethionine methyltransferase that requires only two conserved cysteine residues. Molecular Microbiology.

[ref-28] Irshad S, Xie Z, Mehmood S, Nawaz A, Ditta A, Mahmood Q (2021). Insights into conventional and recent technologies for arsenic bioremediation: a systematic review. Environmental Science and Pollution Research.

[ref-29] Jackson CR, Dugas SL, Harrison KG (2005). Enumeration and characterization of arsenate-resistant bacteria in arsenic free soils. Soil Biology & Biochemistry.

[ref-30] Jamil FN, Hashim AM, Yusof MT, Saidi NB (2022). Analysis of soil bacterial communities and physicochemical properties associated with Fusarium wilt disease of banana in Malaysia. Scientific Reports.

[ref-31] Jia Y, Huang H, Zhong M, Wang FH, Zhang LM, Zhu YG (2013). Microbial arsenic methylation in soil and rice rhizosphere. Environmental Science & Technology.

[ref-32] Kabiraj A, Biswas R, Halder U, Bandopadhyay R (2022). Bacterial arsenic metabolism and its role in arsenic bioremediation. Current Microbiology.

[ref-33] Kabiraj A, Laha A, Panja AS, Bandopadhyay R (2023). *In silico* comparative structural and functional analysis of arsenite methyltransferase from bacteria, fungi, fishes, birds, and mammals. Journal of Genetic Engineering and Biotechnology.

[ref-34] Kaushal M, Tumuhairwe JB, Kaingo J, Richard M, Nakamanya F, Taulya G, Coyne D (2022). Compositional shifts in microbial diversity under traditional banana cropping systems of Sub-Saharan Africa. Biology.

[ref-35] Kumar S, Stecher G, Li M, Knyaz C, Tamura K (2018). MEGA X: molecular evolutionary genetics analysis across computing platforms. Molecular Biology and Evolution.

[ref-36] Kuramata M, Sakakibara F, Kataoka R, Abe T, Asano M, Baba K, Takagi K, Ishikawa S (2015). Arsenic biotransformation by *Streptomyces* sp. isolated from rice rhizosphere. Environmental Microbiology.

[ref-37] Kurth D, Amadio A, Ordoñez OF, Albarracín VH, Gärtner W, Farías ME (2017). Arsenic metabolism in high altitude modern stromatolites revealed by metagenomic analysis. Scientific Reports.

[ref-38] Langille MG, Zaneveld J, Caporaso JG, McDonald D, Knights D, Reyes JA, Clemente JC, Burkepile DE, Vega Thurber RL, Knight R, Beiko RG (2013). Predictive functional profiling of microbial communities using 16S rRNA marker gene sequences. Nature Biotechnology.

[ref-39] Langmead B, Salzberg SL (2012). Fast gapped-read alignment with Bowtie 2. Nature Methods.

[ref-40] Lett MC, Muller D, Lièvremont D, Silver S, Santini J (2012). Unified nomenclature for genes involved in prokaryotic aerobic arsenite oxidation. Journal of Bacteriology.

[ref-41] Li H, Handsaker B, Wysoker A, Fennell T, Ruan J, Homer N, Marth G, Abecasis G, 1000 Genome Project Data Processing Subgroup (2009). The sequence alignment/map format and SAMtools. Bioinformatics.

[ref-42] Li W, Godzik A (2006). Cd-hit: a fast program for clustering and comparing large sets of protein or nucleotide sequences. Bioinformatics.

[ref-43] Li X, Qiao J, Li S, Haggblom MM, Li F, Hu M (2019). Bacterial communities and functional genes stimulated during anaerobic arsenite oxidation and nitrate reduction in a paddy soil. Environmental Science & Technology.

[ref-44] Liu M, Li X, Zhu R, Chen N, Ding L, Chen C (2021). Vegetation richness, species identity and soil nutrients drive the shifts in soil bacterial communities during restoration process. Environmental Microbiology Reports.

[ref-45] Liu J, Su J, Wang J, Song X, Wang H (2022). A case study: arsenic, cadmium and copper distribution in the soil–rice system in two main rice-producing provinces in China. Sustainability.

[ref-46] Liu ZT, Xian WD, Li MM, Liu L, Ming YZ, Jiao JY, Fang BZ, Xiao M, Li WJ (2020). Microvirga arsenatis sp. nov. an arsenate reduction bacterium isolated from Tibet hot spring sediments. Antonie Van Leeuwenhoek.

[ref-47] Lu SG, Xu QF (2009). Competitive adsorption of Cd, Cu, Pb and Zn by different soils of Eastern China. Environmental Geology.

[ref-48] Luo J, Bai Y, Liang J, Qu J (2014). Metagenomic approach reveals variation of microbes with arsenic and antimony metabolism genes from highly contaminated soil. PLOS ONE.

[ref-49] Ma J, Mi Y, Li Q, Chen L, Du L, He L, Lei M (2016). Reduction, methylation, and translocation of arsenic in Panax notoginseng grown under field conditions in arsenic-contaminated soils. Science of the Total Environment.

[ref-50] Martin M (2011). Cutadapt removes adapter sequences from high-throughput sequencing reads. EMBnet Journal.

[ref-51] Marwa N, Singh N, Srivastava S, Saxena G, Pandey V, Singh N (2019). Characterizing the hypertolerance potential of two indigenous bacterial strains (*Bacillus flexus* and *Acinetobacter junii*) and their efficacy in arsenic bioremediation. Journal of Applied Microbiology.

[ref-52] Meharg AA, Meharg C (2021). The pedosphere as a sink, source, and record of anthropogenic and natural arsenic atmospheric deposition. Environmental Science & Technology.

[ref-53] Mirza BS, Sorensen DL, Dupont RR, McLean JE (2017). New arsenate reductase gene (*arrA*) PCR primers for diversity assessment and quantification in environmental samples. Applied and Environmental Microbiology.

[ref-54] Mujawar SY, Vaigankar DC, Dubey SK (2021). Biological characterization of *Bacillus flexus* strain SSAI1 transforming highly toxic arsenite to less toxic arsenate mediated by periplasmic arsenite oxidase enzyme encoded by *aioAB* genes. BioMetals.

[ref-55] Mukhopadhyay R, Rosen BP, Phung LT, Silver S (2002). Microbial arsenic: from geocycles to genes and enzymes. FEMS Microbiology Reviews.

[ref-56] Noulas C, Tziouvalekas M, Karyotis T (2018). Zinc in soils, water and food crops. Journal of Trace Elements in Medicine and Biology.

[ref-57] Nurk S, Meleshko D, Korobeynikov A, Pevzner PA (2017). metaSPAdes: a new versatile metagenomic assembler. Genome Research.

[ref-58] Olmstead LB, Alexander LT, Middleton HE (1930). A pipette method of mechanical analysis of soils based on improved dispersion procedure. Technical Bulletin 170.

[ref-59] Osborne TH, McArthur JM, Sikdar PK, Santini JM (2015). Isolation of an arsenate-respiring bacterium from a redox front in an arsenic-polluted aquifer in West Bengal. Bengal Basin. Environmental Science & Technology.

[ref-60] Osuna-Martínez CC, Armienta MA, Bergés-Tiznado ME, Páez-Osuna F (2021). Arsenic in waters, soils, sediments, and biota from Mexico: an environmental review. Science of the Total Environment.

[ref-61] Qiao JT, Li XM, Hu M, Li FB, Young LY, Sun WM, Huang W, Cui JH (2018). Transcriptional activity of arsenic-reducing bacteria and genes regulated by lactate and biochar during arsenic transformation in flooded paddy soil. Environmental Science & Technology.

[ref-62] Qin J, Rosen BP, Zhang Y, Wang G, Franke S, Rensing C (2006). Arsenic detoxification and evolution of trimethylarsine gas by a microbial arsenite S-adenosylmethionine methyltransferase. Proceedings of the National Academy of Sciences of the United States of America.

[ref-63] Quast C, Pruesse E, Yilmaz P, Gerken J, Schweer T, Yarza P, Peplies J, Glöckner FO (2013). The SILVA ribosomal RNA gene database project: improved data processing and web-based tools. Nucleic Acids Research.

[ref-64] Quéméneur M, Cébron A, Billard P, Battaglia-Brunet F, Garrido F, Leyval C, Joulian C (2010). Population structure and abundance of arsenite-oxidizing bacteria along an arsenic pollution gradient in waters of the Upper Isle River Basin, France. Applied and Environmental Microbiology.

[ref-65] Quinlan AR, Hall IM (2010). BEDTools: a flexible suite of utilities for comparing genomic features. Bioinformatics.

[ref-66] Rosen BP (2002). Biochemistry of arsenic detoxification. FEBS Letters.

[ref-67] Ruppert L, Lin ZQ, Dixon RP, Johnson KA (2013). Assessment of solid phase microfiber extraction fibers for the monitoring of volatile organoarsinicals emitted from a plant–soil system. Journal of Hazardous Materials.

[ref-68] Sabet Aghlidi P, Cheraghi M, Lorestani B, Sobhanardakani S, Merrikhpour H (2020). Analysis, spatial distribution and ecological risk assessment of arsenic and some heavy metals of agricultural soils, case study: South of Iran. Journal of Environmental Health Science & Engineering.

[ref-69] Saltikov CW, Newman DK (2003). Genetic identification of a respiratory arsenate reductase. Proceedings of the National Academy of Sciences of the United States of America.

[ref-70] Schloss PD, Westcott SL, Ryabin T, Hall JR, Hartmann M, Hollister EB, Lesniewski RA, Oakley BB, Parks DH, Robinson CJ, Sahl JW, Stres B, Thallinger GG, Van Horn DJ, Weber CF (2009). Introducing mothur: open-source, platform-independent, community-supported software for describing and comparing microbial communities. Applied and Environmental Microbiology.

[ref-71] Seemann T (2014). Prokka: rapid prokaryotic genome annotation. Bioinformatics.

[ref-72] Shi M, Li J, Zhou Q, Wang G, Zhang W, Zhang Z, Gao Y, Yan S (2020). Interactions between elevated CO_2_ levels and floating aquatic plants on the alteration of bacterial function in carbon assimilation and decomposition in eutrophic waters. Water Research.

[ref-73] Singh N, Ghosh PK, Chakraborty S, Majumdar S (2021). Decoding the pathways of arsenic biotransformation in bacteria. Environmental Sustainability.

[ref-74] Sun S, Jones RB, Fodor AA (2020). Inference-based accuracy of metagenome prediction tools varies across sample types and functional categories. Microbiome.

[ref-75] Sun Y, Polishchuk EA, Radoja U, Cullen WR (2004). Identification and quantification of *arsC* genes in environmental samples by using real-time PCR. Journal of Microbiological Methods.

[ref-76] Tamura K, Nei M, Kumar S (2004). Prospects for inferring very large phylogenies by using the neighbor-joining method. Proceedings of the National Academy of Sciences of the United States of America.

[ref-77] Tapase SR, Kodam KM (2018). Assessment of arsenic oxidation potential of *Microvirga indica* S-MI1b sp. nov. in heavy metal polluted environment. Chemosphere.

[ref-78] Tapase SR, Mawlankar RB, Sundharam SS, Krishnamurthi S, Dastager SG, Kodam KM (2017). Microvirga indica sp. nov. an arsenite-oxidizing Alphaproteobacterium, isolated from metal industry waste soil. International Journal of Systematic and Evolutionary Microbiology.

[ref-79] Tian H, Jing C (2014). Genome sequence of the aerobic arsenate-reducing bacterium *Pantoea* sp. strain IMH. Genome Announcements.

[ref-80] Trapnell C, Williams BA, Pertea G, Mortazavi A, Kwan G, Van Baren MJ, Salzberg SL, Wold BJ, Pachter L (2010). Transcript assembly and quantification by RNA-Seq reveals unannotated transcripts and isoform switching during cell differentiation. Nature Biotechnology.

[ref-81] Vasileiadis S, Coppolecchia D, Puglisi E, Balloi A, Mapelli F, Hamon RE, Daffonchio D, Trevisan M (2012). Response of ammonia oxidizing bacteria and archaea to acute zinc stress and different moisture regimes in soil. Microbial Ecology.

[ref-82] Wang JL, Liu KL, Zhao XQ, Gao GF, Wu YH, Shen RF (2022). Microbial keystone taxa drive crop productivity through shifting aboveground-belowground mineral element flows. Science of the Total Environment.

[ref-83] Wang P, Sun G, Jia Y, Meharg AA, Zhu Y (2014). A review on completing arsenic biogeochemical cycle: microbial volatilization of arsines in environment. Journal of Environmental Sciences.

[ref-84] Xiao KQ, Li LG, Ma LP, Zhang SY, Bao P, Zhang T, Zhu YG (2016). Metagenomic analysis revealed highly diverse microbial arsenic metabolism genes in paddy soils with low-arsenic contents. Environmental Pollution.

[ref-85] Xu C, Zhou T, Kuroda M, Rosen BP (1998). Metalloid resistance mechanisms in prokaryotes. Journal of Biochemistry.

[ref-86] Yan G, Chen X, Du S, Deng Z, Wang L, Chen S (2019). Genetic mechanisms of arsenic detoxification and metabolism in bacteria. Current Genetics.

[ref-87] Yan D, Gellie NJ, Mills JG, Connell G, Bissett A, Lowe AJ, Breed MF (2020). A soil archaeal community responds to a decade of ecological restoration. Restoration Ecology.

[ref-88] Yang YP, Tang XJ, Zhang HM, Cheng WD, Duan GL, Zhu YG (2020). The characterization of arsenic biotransformation microbes in paddy soil after straw biochar and straw amendments. Journal of Hazardous Materials.

[ref-89] Zhang C, Xiao X, Zhao Y, Zhou J, Sun B, Liang Y (2021). Patterns of microbial arsenic detoxification genes in low-arsenic continental paddy soils. Environmental Research.

[ref-90] Zhang SY, Zhao FJ, Sun GX, Su JQ, Yang XR, Li H, Zhu YG (2015). Diversity and abundance of arsenic biotransformation genes in paddy soils from southern China. Environmental Science & Technology.

[ref-91] Zhao FZ, Bai L, Wang JY, Deng J, Ren CJ, Han XH, Yang GH, Wang J (2019). Change in soil bacterial community during secondary succession depend on plant and soil characteristics. Catena.

[ref-92] Zhou D, Jing T, Chen Y, Wang F, Qi D, Feng R, Xie J, Li H (2019). Deciphering microbial diversity associated with *Fusarium* wilt-diseased and disease-free banana rhizosphere soil. BMC Microbiology.

[ref-93] Zhou M, Liu Z, Zhang B, Yang J, Hu B (2022). Interaction between arsenic metabolism genes and arsenic leads to a lose-lose situation. Environmental Pollution.

[ref-94] Zhu L, Ping W, Zhang S, Chen Y, Zhang Y, Zhang J (2021). Description and genome analysis of *Microvirga antarctica* sp. nov. a novel pink-pigmented psychrotolerant bacterium isolated from Antarctic soil. Antonie van Leeuwenhoek.

[ref-95] Zou Q, Wei H, Chen Z, Ye P, Zhang J, Sun M, Huang L, Li J (2023). Soil particle size fractions affect arsenic (As) release and speciation: insights into dissolved organic matter and functional genes. Journal of Hazardous Materials.

