# Supplementary material for: The role of microbiomes in cooperative detoxification mechanisms of arsenate reduction and arsenic methylation in surface agricultural soil
Source: PeerJ. 2024 Oct 30;12:e18383. doi: 10.7717/peerj.18383 (PMC11531259; doi:10.7717/peerj.18383)
Supplement: Supplemental Information 2 [file peerj-12-18383-s002.pdf]

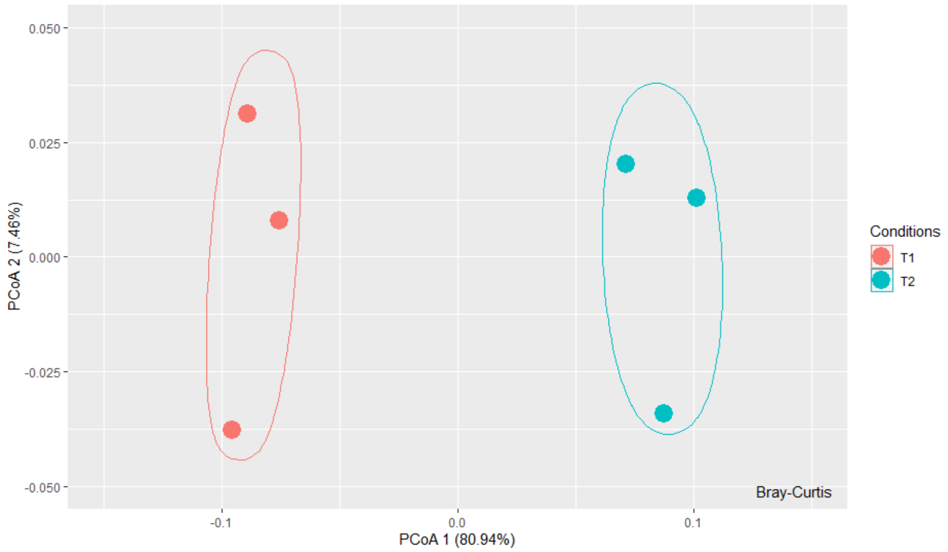

Figure S2. Principal coordinate analysis (PCoA) based on Bray-Curtis distance matrix of soil samples collected from the dry (T1) and wet (T2) seasons with three replications for each season.
