# Supplementary material for: The role of microbiomes in cooperative detoxification mechanisms of arsenate reduction and arsenic methylation in surface agricultural soil
Source: PeerJ. 2024 Oct 30;12:e18383. doi: 10.7717/peerj.18383 (PMC11531259; doi:10.7717/peerj.18383)
Supplement: Supplemental Information 3 [file peerj-12-18383-s003.pdf]

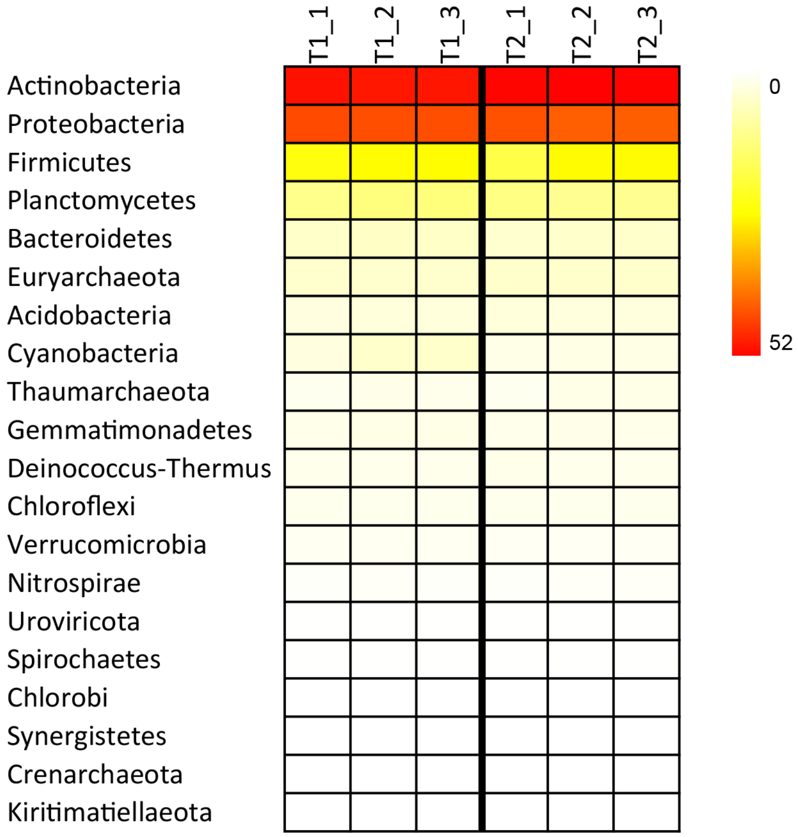

Figure S3. Relative abundance obtained from metagenomic analysis at the phylum level of both the dry season (T1_1, T1_2, and T1_3) and wet season (T2_1, T2_2, and T2_3).
