# Supplementary material for: The role of microbiomes in cooperative detoxification mechanisms of arsenate reduction and arsenic methylation in surface agricultural soil
Source: PeerJ. 2024 Oct 30;12:e18383. doi: 10.7717/peerj.18383 (PMC11531259; doi:10.7717/peerj.18383)
Supplement: Supplemental Information 4 [file peerj-12-18383-s004.pdf]

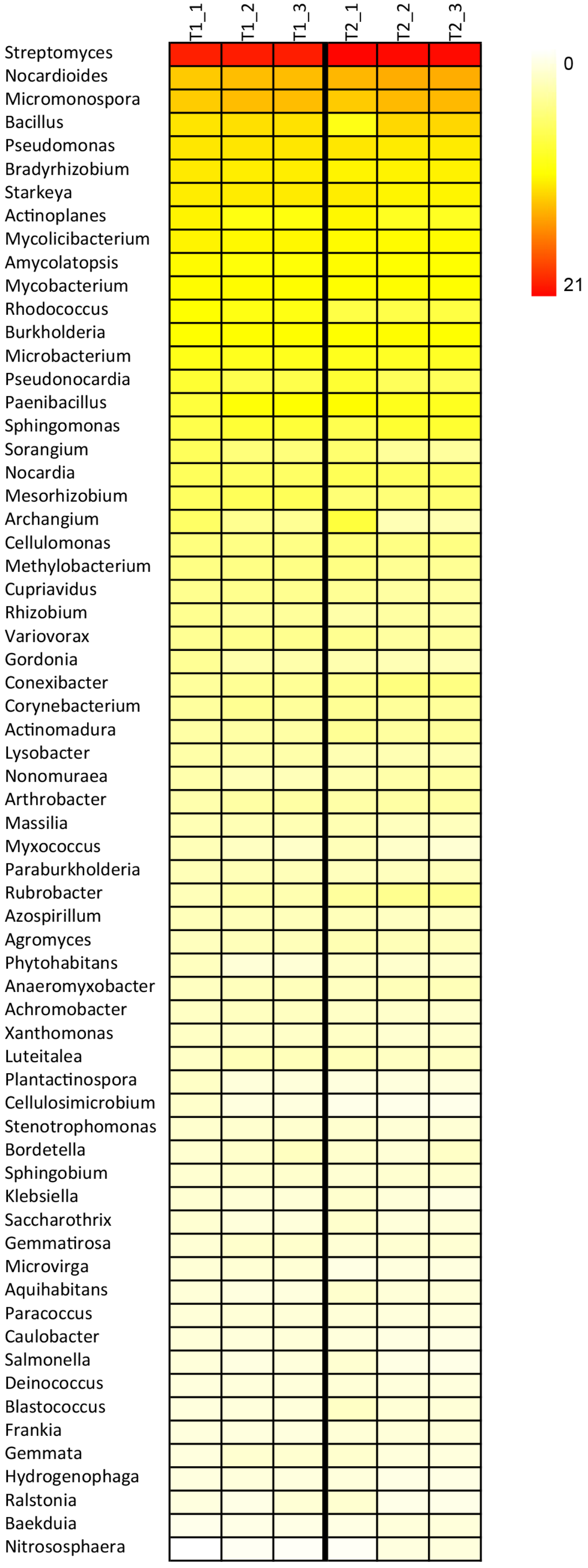
T1_1
T1_2
T1_3
T2_1
T2_2
T2_3
Streptomyces
Nocardioides
Micromonospora
Bacillus
Pseudomonas
Bradyrhizobium
Starkeya
Actinoplanes
Mycolicibacterium
Amycolatopsis
Mycobacterium
Rhodococcus
Burkholderia
Microbacterium
Pseudonocardia
Paenibacillus
Sphingomonas
Sorangium
Nocardia
Mesorhizobium
Archangium
Cellulomonas
Methylobacterium
Cupriavidus
Rhizobium
Variovorax
Gordonia
Conexibacter
Corynebacterium
Actinomadura
Lysobacter
Nonomuraea
Arthrobacter
Massilia
Myxococcus
Paraburkholderia
Rubrobacter
Azospirillum
Agromyces
Phytohabitans
Anaeromyxobacter
Achromobacter
Xanthomonas
Luteitalea
Plantactinospora
Cellulosimicrobium
Stenotrophomonas
Bordetella
Sphingobium
Klebsiella
Saccharothrix
Gemmatirosa
Microvirga
Aquihabitans
Paracoccus
Caulobacter
Salmonella
Deinococcus
Blastococcus
Frankia
Gemmata
Hydrogenophaga
Ralstonia
Baekduia
Nitrososphaera
0
21

Figure S4. Relative abundance obtained from metagenomic analysis at the genus level of both the dry season (T1_1, T1_2, and T1_3) and wet season (T2_1, T2_2, and T2_3).
