# Supplementary material for: The role of microbiomes in cooperative detoxification mechanisms of arsenate reduction and arsenic methylation in surface agricultural soil
Source: PeerJ. 2024 Oct 30;12:e18383. doi: 10.7717/peerj.18383 (PMC11531259; doi:10.7717/peerj.18383)
Supplement: Supplemental Information 5 [file peerj-12-18383-s005.docx]

**Table S1.** Arsenic specific primers used in this study

| Target gene | Primer | Sequence (5´-3´) | Annealing temp. (°c) | Reference |
| --- | --- | --- | --- | --- |
| *aioA* | aoxBM1-2F-ND | CCACTTCTGCATCGTGGGCTGTGGCTA | 53 | Quemeneur *et al*., 2010 |
|  | aoxBM2-1R-ND | GGAGTTGTAGGCGGGCCGGTTGTGGAT |  |  |
| *arrA* | arrA-CVF1 | CACAGCGCCATCTGCGCCGA | 57 | Mirza *et al*., 2017 |
|  | arrA-CVR1 | CCGACGAACTCCYTGYTCCA |  |  |
| *arsC* | smrc-42-f | TCACGCAATACCCTTGAAATGATC | 55 | Sun *et al*., 2004 |
|  | smrc-376-r | ACCTTTTCACCGTCCTCTTTCGT |  |  |
| *arsM* | arsMF1 | TCYCTCGGCTGCGGCAAYCCVAC | 57 | Jia *et al*., 2013 |
|  | arsMR2 | CGWCCGCCWGGCTTWAGYACCCG |  |  |

References

Jia, Y., Huang, H., Zhong, M., Wang, F.H., Zhang, L.M., Zhu, Y.G., 2013. Microbial arsenic methylation in soil and rice rhizosphere. Environ. Sci. Technol. 47, 3141-3148. https://doi.org/10.1021/es303649v.

Mirza, B.S., Sorensen, D.L., Dupont, R.R., McLean, J.E., 2017. New arsenate reductase gene (*arrA*) PCR primers for diversity assessment and quantification in environmental samples. Appl. Environ. Microbiol. 83, e02725-16. https://doi.org/10.1128/AEM.02725-16.

Quéméneur, M., Cébron, A., Billard, P., Battaglia-Brunet, F., Garrido, F., Leyval, C., Joulian, C., 2010. Population structure and abundance of arsenite-oxidizing bacteria along an arsenic pollution gradient in waters of the Upper Isle River Basin, France. Appl. Environ. Microbiol. 76, 4566-4570. https://doi.org/10.1128/AEM.03104-09.

Sun, Y., Polishchuk, E.A., Radoja, U., Cullen, W.R., 2004. Identification and quantification of *arsC* genes in environmental samples by using real-time PCR. J. Microbiol. Methods. 58, 335-349. https://doi.org/10.1016/j.mimet.2004.04.015.
