# Supplementary material for: The role of microbiomes in cooperative detoxification mechanisms of arsenate reduction and arsenic methylation in surface agricultural soil
Source: PeerJ. 2024 Oct 30;12:e18383. doi: 10.7717/peerj.18383 (PMC11531259; doi:10.7717/peerj.18383)
Supplement: Supplemental Information 6 [file peerj-12-18383-s006.docx]

**Table S2.** Summary of the 16S rRNA gene sequence reads during quality control assessment

|  | T1_1 | T1_2 | T1_3 | T2_1 | T2_2 | T2_3 |
| --- | --- | --- | --- | --- | --- | --- |
| Raw reads | 181,851 | 169,056 | 187,258 | 181,955 | 152,505 | 189,101 |
| Filtered reads | 147,585 | 137,031 | 152,454 | 148,082 | 124,326 | 153,597 |
| Denoised reads | 141,663 | 131,755 | 146,401 | 142,533 | 119,245 | 148,239 |
| Merged reads | 117,994 | 111,858 | 119,733 | 117,589 | 98,060 | 125,090 |
| Non-chimeric reads | 104,917 | 99,843 | 104,946 | 103,882 | 86,573 | 111,146 |
