# Supplementary material for: The role of microbiomes in cooperative detoxification mechanisms of arsenate reduction and arsenic methylation in surface agricultural soil
Source: PeerJ. 2024 Oct 30;12:e18383. doi: 10.7717/peerj.18383 (PMC11531259; doi:10.7717/peerj.18383)
Supplement: Supplemental Information 7 [file peerj-12-18383-s007.docx]

**Table S3.** Diversity indexes of soil samples

|  | T1_1 | T1_2 | T1_3 | T2_1 | T2_2 | T2_3 |
| --- | --- | --- | --- | --- | --- | --- |
| Chao1 | 2,181.26 | 2,131.70 | 2,199.91 | 2,006.05 | 1,778.72 | 2,024.03 |
| Simpson’s index | 0.9969 | 0.9966 | 0.9966 | 0.9965 | 0.9957 | 0.9957 |
