# Supplementary material for: The role of microbiomes in cooperative detoxification mechanisms of arsenate reduction and arsenic methylation in surface agricultural soil
Source: PeerJ. 2024 Oct 30;12:e18383. doi: 10.7717/peerj.18383 (PMC11531259; doi:10.7717/peerj.18383)
Supplement: Supplemental Information 8 [file peerj-12-18383-s008.docx]

**Table S4.** Summary of the *aioA*, *arrA*, and *arsM* sequence reads during quality control assessment

|  | T1_*aioA* | T2*_aioA* | T1_*arrA* | T2_*arrA* | T1_*arsM* | T2_*arsM* |
| --- | --- | --- | --- | --- | --- | --- |
| Raw reads (reads) | 119,891 | 54,654 | 138,093 | 56,016 | 87,552 | 56,853 |
| Joint sequence after blastx (reads) | 92,410 | 5,548 | 1,064 | 242 | 54,134 | 9,964 |
| Filtering sequence^1^ (reads) | 49,688 | 3,360 | 498 | 78 | 32,158 | 7,487 |
| OTUs (clusters) | 13,513 | 1,753 | 131 | 30 | 11,507 | 1,803 |

^1^Sequences were filtered with 80% coverage and 80% similarity.
