# Supplementary material for: The role of microbiomes in cooperative detoxification mechanisms of arsenate reduction and arsenic methylation in surface agricultural soil
Source: PeerJ. 2024 Oct 30;12:e18383. doi: 10.7717/peerj.18383 (PMC11531259; doi:10.7717/peerj.18383)
Supplement: Supplemental Information 9 [file peerj-12-18383-s009.docx]

**Table S5.** Abundance of sequences associated with nitrogen metabolism, as determined by 16S rRNA gene sequencing using the PICRUSt2 tool.

| EC number | function  description | T1_1 | T1_2 | T1_3 | T2_1 | T2_2 | T2_3 |
| --- | --- | --- | --- | --- | --- | --- | --- |
| EC:1.7.99.4 | Nitrate reductase | 82155 | 80036 | 79027 | 84969 | 79989 | 82356 |
| EC:1.7.1.15 | Nitrite reductase (NADH) | 63800 | 63813 | 62929 | 65958 | 64224 | 68105 |
| EC:1.7.2.1 | Nitrite reductase (NO-forming) | 10324 | 12771 | 12082 | 14738 | 17941 | 17269 |
| EC:1.7.99.1 | Hydroxylamine reductase | 8038 | 7634 | 8596 | 5941 | 5420 | 6390 |
| EC:1.7.7.1 | Ferredoxin--nitrite reductase | 4249 | 4058 | 4581 | 3939 | 3908 | 3768 |
| EC:1.7.2.2 | Nitrite reductase (cytochrome; ammonia-forming) | 4247 | 4256 | 4464 | 4383 | 3985 | 4630 |
| EC:1.18.6.1 | Nitrogenase | 3316 | 2838 | 3251 | 2633 | 2732 | 2833 |
| EC:1.7.2.4 | Nitrous-oxide reductase | 3155 | 3044 | 3234 | 3244 | 2995 | 3589 |
| EC:1.7.2.5 | Nitric-oxide reductase (cytochrome c) | 1721 | 1706 | 1645 | 1502 | 1373 | 1475 |
| EC:1.7.7.2 | Ferredoxin--nitrate reductase | 1375 | 1290 | 1417 | 879 | 1043 | 1178 |
| EC:1.14.99.39 | Ammonia monooxygenase | 883 | 2791 | 2300 | 3039 | 5266 | 4901 |
| EC:1.14.18.3 | Methane monooxygenase (particulate) | 883 | 2791 | 2300 | 3039 | 5266 | 4901 |
| EC:1.7.2.6 | Hydroxylamine dehydrogenase | 113 | 126 | 141 | 267 | 249 | 262 |
