# Supplementary material for: The role of microbiomes in cooperative detoxification mechanisms of arsenate reduction and arsenic methylation in surface agricultural soil
Source: PeerJ. 2024 Oct 30;12:e18383. doi: 10.7717/peerj.18383 (PMC11531259; doi:10.7717/peerj.18383)
Supplement: Supplemental Information 10 [file peerj-12-18383-s010.docx]

**Table S6.** Summary of the metagenomic sequence reads during quality control assessment.

| Sample ID | T1_1 | T1_2 | T1_3 | T2_1 | T2_2 | T2_3 |
| --- | --- | --- | --- | --- | --- | --- |
| Raw reads (read) | 61,077,982 | 50,356,937 | 47,042,505 | 72,036,454 | 54,517,301 | 47,133,659 |
| Total reads after adapter sequence removal with Q20L100 (read) | 54,705,874 | 45,584,533 | 42,714,252 | 66,261,442 | 49,868,778 | 42,806,116 |
| Total mapped reads (read) | 18,307,778 | 13,950,845 | 12,108,562 | 32,101,269 | 21,821,280 | 17,606,583 |
